# A Comparative Analysis of Color Stability in Direct and Indirect Restorative Materials Following Immersion in a Coffee Solution

**DOI:** 10.1155/ijod/7548302

**Published:** 2025-10-03

**Authors:** Sara Bahrami, Mohammadreza Mokarram, Saba Zamani, Parto Nasrollahi, Soolmaz Heidari, Mehdi Ranjbaran, Sharare Jahangiri, Fahimeh Nouri

**Affiliations:** ^1^Department of Operative Dentistry, Faculty of Dentistry, AJA University of Medical Sciences, Tehran, Iran; ^2^Department of Operative Dentistry, Student Research Committee, School of Dentistry, Qazvin University of Medical Sciences, Qazvin, Iran; ^3^Department of Pediatric Dentistry, Dental Students' Research Committee, School of Dentistry, Isfahan University of Medical Sciences, Isfahan, Iran; ^4^Department of Operative Dentistry, School of Dentistry, Qazvin University of Medical Sciences, Qazvin, Iran; ^5^Non-Communicable Diseases Research Center, Research Institute for Prevention of Non-Communicable Diseases, Qazvin University of Medical Sciences, Qazvin, Iran; ^6^Pediatric Inherited Diseases Research Center, Research Institute for Primordial Prevention of Non-Communicable Disease, Isfahan University of Medical Sciences, Isfahan, Iran

**Keywords:** color stability, restoration, spectroradiometer

## Abstract

**Introduction:**

This study aims to determine the color stability of direct and indirect restorative materials after immersion in coffee solution.

**Materials and Methods:**

A total of 32 blocks (12 mm × 10 mm × 2 mm) were prepared from four tested materials; feldspar ceramic (VITABLOCS Mark II: Vita Zahnfabrik, Germany), hybrid ceramic (Vita Enamic: Vita Zahnfabrik, Germany), indirect composite resin (Gradia Indirect: GC, Japan), and direct composite resin (G-aenial anterior: GC, Japan). The color of all specimens was measured by a spectroradiometer using CIELAB color space before and after thermocycling (5000 cycles) followed by immersion in coffee solution. The color difference (*ΔE*) was calculated by measuring *L*^*∗*^, *a*^*∗*^, and *b*^*∗*^ color values. Data were analyzed using ANOVA and post hoc tests at the significant level of 0.05.

**Results:**

All materials tested exhibited unacceptable color changes after immersion in the coffee solution. The highest value of color change (*ΔE*) was reported for the Gradia Indirect composite, followed by the Vita Enamic and G-aenial and VITABLOCS Mark II groups, respectively. However, no significant differences were observed between any of the groups (*p* = 0.058). G-aenial showed the most visual discoloration.

**Conclusions:**

The study demonstrated that there were no statistically significant differences in color change between the various groups following immersion in coffee. Additionally, the color change exceeded the clinically acceptable limit in all samples. It is noteworthy that differences in the initial color values (*L*^*∗*^, *a*^*∗*^, and *b*^*∗*^) resulting from the different compositions and structures of the materials may influence visual perception.

## 1. Introduction

The color stability of dental restorations is of particular importance and is one of the key factors influencing the success of dental treatments. Dental resin composites are susceptible to water absorption and color changes due to their resin matrix. In contrast, most dental ceramics can exhibit greater color stability due to the absence of a resin matrix. However, the color stability of dental restorations is influenced by a number of internal and external factors. From an external perspective, oral hygiene, smoking, and the consumption of foods containing dyes or substances that may cause deterioration of the restoration structure and consequently provide the conditions for stain penetration should be considered. Among the internal parameters, the presence of unreacted monomers and their subsequent hydrolysis are worthy of mention. Furthermore, the presence of photoinitiators that do not participate in the curing process may also result in color changes. The characteristics of the resin matrix and fillers used, the degree of polymerization, and the amount of water absorption can also be effective [1]. However, even in dental ceramics, color changes of restoration have been reported over time. In addition to external factors, internal factors such as ceramic hardness and aging are also effective in inducing color changes in indirect ceramic restorations [2]. The quality of finishing and polishing has consistently been identified as a significant factor influencing the color stability of dental restorations [3, 4].

In the present study, in addition to a direct dental composite, an indirect composite and a hybrid ceramic have also been investigated. Despite significant advancements in the field of direct dental composites, indirect composites continue to occupy a position in the dental market due to their inherent advantages. These materials exhibit reduced shrinkage and subsequent microleakage, as well as enhanced mechanical properties. While the presence of a resin matrix may still result in color changes of these restorations, achieving a higher degree of conversion in these composites may potentially reduce the extent of such changes. The curing of these laboratory composites is typically achieved through light or heat [5, 6]. Achieving a higher degree of conversion is recognized as one of the key factors influencing the color stability of resin-based restorations [6]. In recent years, materials known as hybrid ceramics have entered the dental market. These materials have a Young's modulus close to that of dentin and can distribute stress better. They are divided into two groups: resin nanoceramic and polymer-infiltrated ceramic network (PICN) [7]. They have a similar formulation to dental composites. The laboratory process of these ceramics is only CAD/CAM (computer-aided design/computer-aided manufacturing), without the need for additional heat treatment in the laboratory. The resin matrix part of these ceramics is polymerized by the use of powerful heating systems [8]. The presence of a resin matrix can still be involved in the color change [9].

The present study compared the color stability of three different resin-based materials used in the fabrication of dental restorations (including a direct resin composite, an indirect composite, and a PICN hybrid ceramic) after immersion in a coloring solution (coffee). In this study, a feldspathic ceramic was used as a control group. This ceramic has no resin content, which may be effective in reducing the extent of color change [10]. The purpose of using different systems, from direct composite to indirect ceramic, was to evaluate dental restorations with different percentages of resin and ceramic, an issue that is less visible in existing studies.

## 2. Materials and Methods

A total of 32 blocks of four tested materials (eight in each group) were prepared with dimensions of 12 mm × 10 mm × 2 mm (length: 12 mm, width: 10 mm, thickness: 2 mm). The materials utilized in the present study are detailed in Table 1.

Prefabricated molds of the same dimensions as the ceramic samples were used to fabricate composite blocks. The composite was placed in the mold and a Mylar strip was placed on the surface of the composite, followed by curing.

G-aenial was cured from both sides for 40 s with Valo grand light (Ultradent, USA, 395–480 nm, 1000–1100 mW/cm^2^). The initial curing of Gradia indirect was performed with the Steplight SL-i unit (GC, Japan) containing a 180 W halogen lamp for 10 s. Final curing was performed with a power of 1500 mW/cm^2^ for 3 min (Labo Light LV-III, GC Japan, 140 W, 440–480 nm) [11–13]. Subsequently, polishing was performed with polishing wheels (EVE Diacomp Plus TWIST, Eve Ernest Vetter GmbH, Germany) with a rough to soft sequence for 20 s by each wheel.

The VITABLOCS Mark II and Vita Enamic ceramics were cut to the intended dimensions (12 mm × 10 mm × 2 mm) with a low speed under water cooling conditions (Mecatom 210 A, Presi, France). Subsequently, the surface of the ceramic samples was polished with a ceramic polishing wheel (DiapolEve Twist, Eve Ernest Vetter GmbH, Germany).

Following preparation, the samples were maintained in distilled water at 37°C for 24 h prior to the commencement of the experiment. The initial color evaluation was conducted using a spectroradiometer (CS-2000, Konica Minolta, Japan), which employs a noncontact measuring range as low as 0.0005 cd/m^2^ and a wavelength range of 380–780 nm. The color parameters (*L*^*∗*^, *a*^*∗*^, *b*^*∗*^) were determined by a single operator at 25 ± 4°C and 45% humidity under D65 illuminant and an observer angle of 2° on a white background, based on the CIE *L*^*∗*^*a*^*∗*^*b*^*∗*^ color space. Subsequently, all samples were subjected to 5000 thermal cycles in distilled water at temperatures of 5 and 55°C, with a transfer interval of 10 s and a holding time of 15 s [14]. A solution of coffee (3 g of Nescafé Classic, produced by Nestlé Suisse SA, Vevey, Switzerland) and distilled water (100 cc) was prepared for analysis. The samples were immersed in the coffee solution for a period of 30 min, which is twice the duration typically required for the consumption of coffee. This process was repeated three times a day [15] for a period of 4 weeks. This protocol was selected with the objective of facilitating a more pronounced color change. Following each immersion period, the samples were rinsed with distilled water and maintained in artificial saliva (pH: 6.9, 1000 mL, 1.2 g chloride potassium, 0.84 g sodium chloride, 0.26 g dipotassium phosphate, and 0.14 g calcium chloride dehydrate) [16] until they were immersed again. To simulate the oral cavity environment, the samples were brushed with a toothbrush for 120 s twice a day [17]. The coffee solution was replaced by a new solution every 48 h. After 4 weeks, the samples were removed from the solution, washed with saline, and dried. A second examination was conducted under the spectroradiometer to ascertain the *L*^*∗*^, *a⁣*^*∗*^, *b⁣*^*∗*^ parameters. Thereafter, the *ΔL*, *Δa*, and *Δb* values were calculated, and the *ΔE* value was determined using the following formula:

  $$\begin{array}{c} ∆E^{*}={\left[{\left(∆L^{*}\right)}^{2}+{\left(∆a^{*}\right)}^{2}+{\left(∆b^{*}\right)}^{2}\right]}^{1/2} \end{array}$$

The *L*^*∗*^ parameter represents the brightness or value, which ranges from black (0) to white (100). The coordinate *a*^*∗*^ represents green (−) to red (+), the *b*^*∗*^ coordinate represents blue (−) to yellow (+), and Δ*E* represents the total color change of the samples following thermocycling and immersion processes.

## 3. Statistical Methods

The data analysis was conducted using the IBM SPSS Statistics software, version 25 (Armonk, NY). One-way ANOVA was employed to compare color stability between groups, and post hoc tests were used for pair-wise comparisons. Evaluation of color values (*L*^*∗*^, *a*^*∗*^, and *b*^*∗*^) within each group before and after the intervention was done by paired sample *t*-test. The significance level of the tests was set at *p*  < 0.05.

## 4. Results

Significant differences were identified between the groups with regard to the *Δa* and *Δb* color values, as detailed in Table 2. In terms of *a*^*∗*^ color value, Gradia indirect showed the greatest change compared to the other groups. Significant differences were observed among the other groups, with the exception of Vita Enamic, which did not show statistically significant differences from VITABLOCS Mark II and G-aenial.

The greatest change in *b*^*∗*^ color value was observed in the G-aenial group. Apart from Vita Enamic, which did not show statistically significant differences with VITABLOCS Mark II and Gradia indirect composite, a significant difference was observed between the remaining groups.

With regard to ΔL color value, no statistically significant difference was identified between the groups (Table 2). Nevertheless, the most changes were measured in the Vita Enamic and Gradia indirect composite. Prior to immersion, the spectroradiometry revealed that the G-aenial direct composite exhibited a notable distinction from the other groups, particularly with regard to *a*^*∗*^ coordinate. The mean *a*^*∗*^ color value before immersion for G-aenial was 7.4 ± 0.26, which was significantly higher (*p*  < 0.05) than that of the other three groups (0.41 ± 0.2 for Gradia Indirect, 0.5 ± 0.24 for VITABLOCS Mark II, and 3.55 ± 0.17 for Vita Enamic). Statistical evaluation of the color values (*L*^*∗*^, *a*^*∗*^ and *b*^*∗*^) within each group before and after the intervention was also performed. Both the Gradia indirect and the G-aenial showed significant changes in all parameters. For the ceramics, significant changes were observed only in the *b*^*∗*^ color value (Table 2).

The highest *ΔE* value was associated with the Gradia indirect composite, followed by Vita Enamic and G-aenial composite. In contrast, the lowest *ΔE* value was reported for VITABLOCS Mark II. However, these differences were not statistically significant (Table 3).

**Figure 1:**
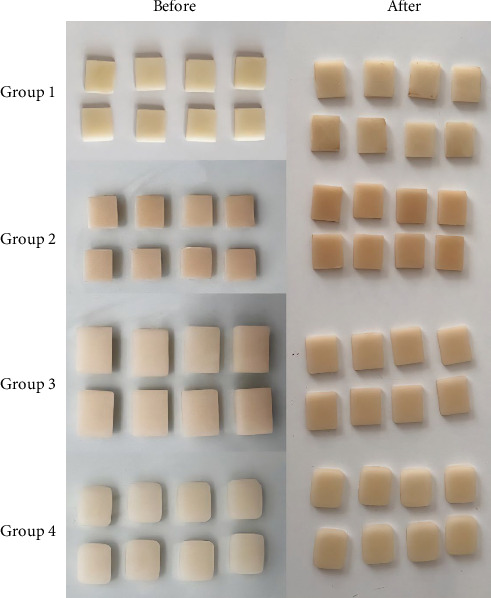
Visual evaluation of different groups before and after immersion in coffee. Group 1: Gradia indirect, Group 2: G-aenial, Group 3: Vita Enamic, and Group 4: VITABLOCS Mark II.

The visual changes of the samples before and after immersion in coffee are shown in Figure 1. Visual evaluation of the samples showed more changes in the resin-based samples. G-aenial showed the most visual discoloration, which is not consistent with the spectroradiometric results. Considering that *ΔE* > 3.3 is considered clinically unacceptable, the discoloration of all study samples was clinically unacceptable.

## 5. Discussion

The present study evaluated the color changes in composite and ceramic samples following thermocycling and immersion in coffee. One of the most frequently utilized methodologies for modeling the physiological aging of dental materials is the application of thermocycling. This process can result in the formation of surface roughness, which, in turn, renders the restoration more susceptible to color alteration [18, 19]. Coffee has been demonstrated to exert a pronounced influence on the color change and staining of restorative materials. Coffee contains tannins and pigments that produce yellow, brown, and red colors [20]. Therefore, in this study, thermocycling and immersion in coffee were used to simulate the oral environment.

In this study, the finishing and polishing tools belonging to a company, which were manufactured separately for each material, were employed. Some previous studies have suggested a potential correlation between surface roughness and color changes [21, 22]. Consequently, an effort was undertaken to achieve a smooth surface across all groups.

The results indicated no statistically significant difference in *ΔE* between any of the studied groups. However, the amount of color change was found to be lower in VITABLOCS Mark II than in the other groups. This finding is consistent with the results of the study by Saba et al. [23], which showed that the total color change of VITABLOCS Mark II was significantly less than that of Vita Enamic after 28 days of immersion in coffee. This finding can be attributed to the absence of a resin matrix in the structure of this ceramic. This material is a feldspar glass–ceramic with leucite crystals and exhibits reduced susceptibility to water sorption and color change in comparison to composites and hybrid ceramics, which contain a resin matrix [24]. The presence of 35% unreacted carbon double bonds in the resin matrix of polymer structures has been observed to cause a significant color change. However, the precise relationship between the amount of unreacted double carbon and the extent of color change remains a topic of debate among researchers [25, 26].

In resin composite materials, color change is influenced by both external factors (external coloring materials, such as coffee) and internal factors related to composite structure (composite polymerization quality, resin matrix composition, amount and distribution of filler, type of initiator, filler/matrix interface, etc.) [1]. For example, UDMA (urethane dimethacrylate) has been demonstrated to exhibit superior color stability compared to monomers such as TEGDMA (triethylene glycol dimethacrylate) and Bis-GMA (bisphenol A-glycidyl methacrylate), which is attributed to its reduced water sorption capacity [27]. UDMA has lower water sorption compared to Bis-GMA and TEGDMA, which may result in more color stability [27, 28].

The color change of the G-aenial direct composite was less than that of the indirect composite and the hybrid ceramic used in this study. However, this difference was not statistically significant. This may be due to the absence of Bis-GMA and the use of UDMA as the main monomer. In addition, the manufacturer has not indicated whether or not TEGDMA is present as a comonomer in the composition of this composite [14]. The manufacturer of Vita Enamic did not mention the presence of Bis-GMA in this hybrid ceramic [29]. However, the presence of this monomer was detected in the study by Mourouzis et al. [30]. The presence of the more hydrophilic TEGDMA monomer (compared to UDMA), despite the high weight percentage of the ceramic component, may be a reason for the color change of this hybrid ceramic. According to the manufacturer, the Vita Enamic manufacturing process uses controlled and precise curing by heat and pressure, resulting in high-quality blocks [29]. However, another probable cause for the color change of the hybrid ceramic may be the possibility of breaking the chemical bond at the ceramic/polymer interface [28], which should be investigated in further studies.

Paolone et al. [31] reported more discoloration with hybrid ceramics than with other ceramics. However, another study showed that Vita Enamic has less discoloration than VITABLOCS Mark II [32]. One of the most important reasons for the difference in these results could be the different techniques used to measure the color change, as well as the difference in shade and translucency of the ceramics studied.

The findings of the present study indicated that the greatest color change was associated with Gradia indirect composite. Despite the use of two curing stages and a longer curing time, this composite exhibited greater color change than the G-aenial direct composite. Although indirect composites are thought to demonstrate superior color stability compared to direct composites due to the potential for more precise control over the curing process, improved polymerization quality and degree of conversion, studies conducted on these composites have yielded conflicting results [33]. A number of studies have demonstrated notable color changes of indirect resin composites [34, 35]. It seems that a number of factors, including the composition of the material, are involved in these findings. However, information regarding the composition of the Gradia indirect composite is currently unavailable. Furthermore, the difference in *L*^*∗*^, *a*^*∗*^, and *b*^*∗*^ color values between the groups prior to immersion, as well as their different trend of changes following immersion in coffee (which is influenced by the shade and translucency of the material), can yield entirely different results [33]. In summary, the three parameters *L*^*∗*^, *a*^*∗*^ and *b*^*∗*^ are influenced by three main factors: the coffee, the composition, and the shade of the material [20, 36].

All materials used in this study demonstrated a positive shift in *a*^*∗*^ color value (tendency to redness) and also exhibited modifications in *b*^*∗*^color value in a negative direction (tendency to blue and diminished yellowness), with the exception of VITABLOCS Mark II. The observed redness in the groups containing polymers can be attributed to the presence of by-products produced by the amine accelerator, which has been demonstrated to create a red–brown color following heating (e.g., thermocycling). It is well documented that the reduction in the value of parameter *b⁣*^*∗*^ (less yellowness) in dental composites following the curing process is due to the loss of yellow pigmentation in camphorquinone. Nevertheless, incomplete polymerization may result in residual camphorquinone, which can impart a yellow hue [36–40]. Alternatively, the residual yellow hue may be attributed to the oxidation of unreacted carbon double bonds [41]. The findings of the present study indicated that the parameter *b*^*∗*^ in G-aenial direct composite, Gradia indirect composite, and Vita Enamic ceramic exhibited a reduction in yellowness following thermocycling and immersion in coffee. The observed changes in parameter *b*^*∗*^, whether an increase or decrease, can be attributed to a number of factors. These include the unique composition of each material, thermal shock, and physical and chemical changes that occur during the thermocycling process. These factors can affect the concentration of camphorquinone. The thermocycling process has the potential to influence water absorption and, consequently, may lead to material degradation [34]. Given the inherent compositional and structural differences among materials, it is reasonable to conclude that the observed changes in color parameters will not be uniform.

The two ceramics evaluated in this study showed less changes in *b*^*∗*^ color value due to the absence of photoinitiator. Regarding the color shift toward redness and the increase in yellowness in feldspar ceramics, it should be mentioned that materials with higher translucency are subject to more staining (toward yellowness and redness) after immersion in colored solutions [42, 43]. All materials in this study showed a significant decrease in brightness after aging. The darkening of the samples after immersion in coffee has been associated with a decrease in value, a phenomenon that has been previously documented [44].

In terms of visual evaluation, all restorative materials exhibited a perceptible color change, which is in alignment with the findings of spectroradiometry. Nevertheless, in contrast to the findings from spectroradiometry, the observed color change of the G-aenial composite was greater than that of the other materials, which may be attributed to the notable difference between the initial coordinate *a*^*∗*^ of this material and those of the other materials. The elevated *a*^*∗*^ and *b*^*∗*^ coordinates have the potential to negatively impact the perceived color [45].

## 6. Conclusions

The present study showed no statistically significant difference in *ΔE* between the studied groups after coffee immersion. The *ΔE* value after 28 days of immersion in coffee solution was higher than the clinically acceptable limit in all groups. However, differences in the primary coordinates (*L*^*∗*^, *a*^*∗*^, and *b*^*∗*^) caused by different intrinsic factors for each material may affect visual perception.

## Figures and Tables

**Table 1:** Properties of the materials used in the present study are listed.

Group	Type	Manufacturer	Composition	Color
Vita Enamic	Polymer infiltrated ceramic network	Vita Zahnfabrik, Bad Sackingen Germany	UDMA and TEGDMA (14 wt%), feldspathic ceramic enriched with aluminum oxide (86 wt%)	2M2

VITABLOCS Mark II	Feldspar ceramic	Vita Zahnfabrik, Bad Sackingen Germany	Fine-particle feldspar ceramic mill block, Al_2_O_3_ - SiO_2_ - Na_2_O - K_2_O	2M2

Gradia indirect	Microfilled indirect resin composite	GC, Tokyo, Japan	UDMA, methacrylate monomers, inorganic and prepolymerized fillers: 75 wt%, photoinitiator, catalyst	E2

G-aenial anterior	Microhybrid-direct resin composite	GC, Tokyo, Japan	UDMA, methacrylate monomers, silica and prepolymerized strontium and lanthanoid fluoride containing fillers: 76 wt%, pigments, camphorquinone, catalyst	A2

Abbreviations: TEGDMA, triethylene glycol dimethacrylate; UDMA, urethane dimethacrylate.

**Table 2:** Evaluation of changes in color values after immersion in coffee.

Group		Mean	Std. deviation	*p*-Value (within each group)	*p*-Value (overall)
*ΔL*	Gradia Indirect	−7.15	2.36	<0.001	0.051
	G-aenial A2	−5.78	1.85	<0.001	
	VITABLOCS Mark II	−5.01	1.36	0.044	
	Vita Enamic	−7.31	1.56	<0.001	

*Δa*	Gradia Indirect	1.71	0.45	0.008	<0.001
	G-aenial A2	0.40*⁣*^*∗*^	0.31	<0.001	
	VITABLOCS Mark II	1.14•	0.38	<0.001	
	Vita Enamic	0.89*⁣*^*∗*^•	0.29	<0.001	

*Δb*	Gradia Indirect	−1.07*⁣*^*∗*^	1.23	0.081	<0.001
	G-aenial A2	−2.92	0.66	<0.001	
	VITABLOCS Mark II	0.41•	0.57	<0.001	
	Vita Enamic	−0.17*⁣*^*∗*^•	0.55	0.403	

*Note: p*-Value within each group is related to the statistical comparison for each color value before and after immersion within the groups. *p*-Value overall is related to the comparisons between groups. Values marked with the same superscript symbol “*⁣*^*∗*^ and •” are not statistically different (*p* > 0.05).

**Table 3:** Color changes observed in different groups.

Groups		Mean	Std. deviation	*p*-Value
*ΔE*	Gradia Indirect	7.51	2.40	0.058
	G-aenial	6.56	1.71	
	VITABLOCS Mark II	5.19	1.40	
	Vita Enamic	7.39	1.56	

*Note:* There were no significant differences between the groups in pair-wise comparisons (*p* < 0.05).

## Data Availability

The data that support the findings of this study are available from the corresponding author upon reasonable request.
